# Function and Modulation of Type I Interferons during Respiratory Syncytial Virus Infection

**DOI:** 10.3390/vaccines8020177

**Published:** 2020-04-10

**Authors:** Laura M. Stephens, Steven M. Varga

**Affiliations:** 1 Interdisciplinary Graduate Program in Immunology, University of Iowa, Iowa City, IA 52242, USA; laura-stephens@uiowa.edu; 2 Department of Microbiology and Immunology, University of Iowa, Iowa City, IA 52242, USA; 3 Department of Pathology, University of Iowa, Iowa City, IA 52242, USA

**Keywords:** respiratory syncytial virus, interferon-α, interferon-β, type I interferons, pDCs, vaccine, neonatal

## Abstract

Respiratory syncytial virus (RSV) is the leading cause of lower respiratory infections in infants and young children, accounting for an estimated 3 million hospitalizations annually worldwide. Despite the major health burden, there is currently no licensed RSV vaccine. RSV is recognized by a range of cellular receptors including both toll-like receptors (TLR) and retinoic acid-inducible gene-I-like receptors (RIG-I). This interaction initiates signaling through mitochondrial antiviral signaling (MAVS) and interferon regulatory factor (IRF) proteins, resulting in the induction of type I interferons (IFN). Early viral control is mediated by either IFN-α or IFN-β signaling through the IFN receptor (IFNAR), inducing the production of antiviral interferon-stimulating genes (ISGs). Type I IFNs also initiate the early production of proinflammatory cytokines including interleukin 6 (IL-6), tumor necrosis factor (TNF), and IFN-γ. Type I IFN levels correlate with age, and inadequate production may be a critical factor in facilitating the increased RSV disease severity observed in infants. Here, we review the current literature on the function of type I IFNs in RSV pathogenesis, as well as their involvement in the differential immune responses observed in infants and adults.

## 1. Introduction

Respiratory syncytial virus (RSV) is the leading cause of lower respiratory infections in infants and young children, accounting for approximately seven percent of deaths in children less than one year of age [1]. Globally, RSV causes 34 million new infections each year, resulting in nearly 3 million hospitalizations [2]. RSV also produces a major economic burden, accounting for more than $300 million in annual medical costs in the United States alone [3]. RSV reinfection is common in children, and adults remain susceptible to repeated infections due to short-lived and incomplete protective immunity following natural infection [4,5]. Despite the critical need for one, there is currently no licensed RSV vaccine.

RSV is a single-stranded, negative-sense RNA virus of the *Pneumoviridae* family. The RSV genome is approximately 15.2 kb in size, and encodes ten genes that transcribe 11 proteins [6]. The RSV matrix (M) protein functions to mediate the assembly of new virions [7]. The nucleocapsid (N), phosphoprotein (P), large polymerase (L), M2-1, and M2-2 proteins make up the transcriptional and replication machinery for RSV [8]. Attachment and fusion of RSV to host cells is mediated by the attachment (G) and fusion (F) glycoproteins located on the surface of the viral membrane, and the small hydrophobic (SH) protein functions as a viroporin to facilitate release of new virions [9,10]. Finally, the nonstructural (NS) proteins 1 and 2 work independently and cooperatively to suppress the antiviral type I interferon (IFN) response in RSV-infected cells [11].

IFNs play a major role in initiating early antiviral responses. Type I IFNs are produced by many cell types including dendritic cells (DCs), epithelial cells, and alveolar macrophages following RSV infection [12,13]. The induction of type I IFNs plays a critical role early during RSV infection [14]. Knockout mouse models for IFN-α/β, as well as downstream signaling components mitochondrial antiviral signaling (MAVS) or retinoic acid-inducible gene-I (RIG-I) have failed to control viral replication [15,16,17]. Binding of type I IFNs to the IFN receptor (IFNAR) leads to the production of numerous interferon-stimulating genes (ISGs) that perform both antiviral and proinflammatory roles [18]. Here, we review the current literature on the role of type I IFNs in the pathogenesis of RSV as well as their contribution to the distinct immune responses observed in infants and adults. The critical antiviral effects of these cytokines, as well as their impact on adaptive immunity, make them attractive targets for generating long-lasting protective immunity against RSV.

## 2. Type I IFN and RSV

### 2.1. The Role of Type I IFN and the Innate Immune Response to RSV

Type I IFNs are a class of related cytokines that differ based on their structure and expression patterns [19]. They include many subtypes of IFN-α (13 in humans) and one IFN-β. There are additional family members that have cell-type and species-specific expressions and will not be discussed in this review. The initiation of type I IFN production begins with the recognition of viral proteins and/or replication products by cytoplasmic and/or surface pattern-recognition receptors (PRRs) [20]. Toll-like receptors (TLRs) are expressed both on the plasma membrane and in the membranes of endosomes, and sense an array of pathogen-derived shared molecules [21]. RIG-I-like receptors, including RIG-I and melanoma differentiation-associated protein 5 (MDA5), are found in the cytoplasm and recognize intracellular viral replication products [22]. Sensing of RSV by TLRs, RIG-I, and/or MDA5 initiates early type I IFN production.

RSV is most commonly detected by TLR2, TLR4 and TLR6, as well as the RIG-I-like receptors [15,23,24]. TLR4 interacts with the RSV F protein to activate the innate immune response and downstream nuclear factor kappa-light-chain-enhancer of activated B cells (NF-κB) activation [24,25]. Treatment of adult peripheral blood mononuclear cells (PBMCs) with CD14-blocking antibodies, as well as a knockout mouse model, identified CD14 as an essential co-factor for TLR4 recognition of RSV F [25]. There is also a potential role for a complex composed of TLR4, CD14, and the accessory protein MD-2 in the recognition of RSV F [26,27]. Defects in TLR4 are linked to severe RSV-induced disease in high-risk premature infants, and PBMCs isolated from these children produce diminished levels of interleukin 8 (IL-8), tumor necrosis factor (TNF), and IFN-α/β when infected with RSV in vitro [28,29,30]. Similarly, TLR2 and TLR6-deficient mice are impaired in their early production of IL-6 and type I IFNs, suggesting that TLR recognition of RSV promotes early initiation of inflammatory responses [24]. Additionally, TLR2, TLR6, and TLR4-deficient mice all exhibited high RSV titers in the lungs post-infection compared to wildtype (WT) mice, demonstrating the in vivo role of TLR-mediated innate sensing in viral clearance [23,24,31]. These data indicate that TLR recognition of RSV is important for both viral clearance and promoting early proinflammatory cytokine production.

Cytoplasmic RIG-I-like receptors are present at low levels in all cells and sense double-stranded RNA and 5’-triphosphate RNA produced during viral infections [16,32]. Interaction between RIG-I and RSV helps initiate the antiviral response, as siRNA knockdown of RIG-I in vitro significantly reduces IFN-β and ISG-15 production at early time points (5–9 hours) post-infection [15,33]. Additionally, loss of function mutations in interferons induced with helicase C domain 1 (IFIH1), which encodes a RIG-I-like receptor involved in viral sensing, is associated with enhanced susceptibility to RSV bronchiolitis in children due to an inability to induce IFN-β production [34]. However, siRNA knockdown of either RIG-I or MDA5 does not impact RSV growth in either A549 or Vero epithelial cells [33]. This suggests that RIG-I-like receptors are necessary for early type I IFN production in response to RSV, but that other sensing mechanisms may be more important for promoting viral clearance.

Following recognition of RSV by TLRs or RIG-I-like receptors, the proteins bind to the adaptor MAVS via caspase activation and recruitment domains (CARD), subsequently recruiting various TNF receptor-associated factor (TRAF) family members [35,36]. Depending on the TRAF that binds, downstream signaling leads to one of two outcomes: phosphorylation of interferon regulatory factor (IRF)3 and IRF7, or activation of IκB kinase (IKK) and NF-κB [37]. The activated transcription factors translocate to the nucleus, where they bind the type I IFN promoters and induce transcription of IFN-α and IFN-β [35]. MAVS, also known as IFN-β promoter stimulator 1 (IPS-1), is critical for the production of type I IFN in response to RSV. In the absence of MAVS, mice produced nearly undetectable levels of IFN-α and IFN-β in both serum and bronchoalveolar lavage (BAL) fluid at day 8 post-RSV infection, and exhibited increased neutrophil recruitment in the lungs [16]. MAVS-deficient mice also exhibit increased viral titers and mRNA levels of the RSV F and N proteins on day four post-infection [12,16,17,38]. However, normal clearance of the infection is achieved by day 9, further emphasizing the importance of MAVS in the early control of RSV replication. Overall, MAVS signaling plays a nonredundant role in the production of proinflammatory cytokines including type I IFNs, and in establishing an antiviral environment early after RSV infection.

In response to RSV, IFN-α production has been measured in epithelial cells, fibroblasts, conventional DCs (cDCs), lung macrophages, and plasmacytoid DCs (pDCs) in vitro [12,39,40,41,42]. However, in vivo models suggest that type I IFNs are produced primarily by pDCs, epithelial cells, and alveolar macrophages [12,13,43,44]. In WT mice, IFN-α is produced by epithelial cells and pDCs in the lungs as early as 24 hours post-RSV infection [39]. Administration of anti-120G8 or a blood dendritic cell antigen 2-diphtheria toxin receptor (BDCA-2-DTR) mouse model to deplete pDCs in vivo abolished IFN-α protein levels [13,45]. pDC depletion also increased viral titers in the lungs on days 4 and 6 post-infection, an effect that could be rescued by the addition of recombinant IFN-α [13,46]. While these studies support an important role of pDCs in the type I IFN response against RSV and the subsequent priming of the adaptive response, one study using plasmacytoid dendritic cell antigen-1 (PDCA-1) antibody treatment to deplete pDCs observed no difference in either IFN-α or IFN-β production in the lungs of RSV-infected WT mice [39]. These apparent discrepancies may be a consequence of the various depletion methods used in each study, however, further experiments are needed to unravel the role of the various type I IFN-producing cell populations in RSV.

Due to the transient production of type I IFNs, their presence can be hard to detect in vivo, facilitating the need for reporter viruses and modified mouse models. These experimental tools, while useful, may not accurately represent what occurs in situ in mice and humans and may explain the inconsistent conclusions found in the literature. Goritzka et al. (2015) utilized a reporter virus that only expressed green fluorescent protein (GFP) in cells with active transcription of IFN-α6 [12]. IFN-α6 is just one of many IFNs that are produced following RSV infection and the narrow focus could, in part, explain why they observe production only within alveolar macrophages. Jewell et al. observed IFN-α4 production in both lung epithelial cells and pDCs, similarly demonstrating a cell-specific preference for production of one IFN-α family member [39]. Finally, the finding that DCs produce the majority of IFN-β in an IFN-β/YFP reporter mouse model suggests that production of IFN-α and IFN-β in response to RSV may occur in different cell types [45]. Overall, many studies have reported type I IFN production by specific cell populations, suggesting that multiple cell types, rather than a single major population, likely contribute to the type I IFN response against RSV in vivo.

All type I IFNs utilize a common cell surface receptor known as the IFN-α receptor (IFNAR), consisting of IFNAR1 and IFNAR2 [47]. Type I IFN binding to IFNAR on any nucleated cell leads to activation of the janus kinase (JAK)/signal transducer and activator of transcription (STAT) signaling cascade and downstream induction of numerous ISGs [18]. During RSV infection, IFNAR signaling and ISGs mediate inflammation by driving the production of critical proinflammatory cytokines. In the absence of IFNAR1, mice fail to produce IFN-α/β, IFN-γ, IL-6, TNF, and CXCL10 in the lungs and BAL in response to RSV [18,38]. Likewise, IFNAR-deficient primary murine alveolar macrophages infected with RSV in vitro produce negligible IFN-α/β compared to WT cells [38]. Type I IFN signaling also contributes to viral control, as IFNAR-deficient mice exhibit increased viral load and weight loss following RSV challenge compared to WT controls [18]. Thus, IFNAR-dependent signaling is required for the antiviral and proinflammatory functions of type I IFNs during RSV infection.

ISGs are critical for initiating an antiviral response, and encode many proteins that limit viral replication. Cyclic guanosine monophosphate–adenosine monophosphate (GMP-AMP) synthase (cGAS) interferes with RSV infectivity in vitro, potentially through the inhibition of IRF3 [48]. The interferon-inducible transmembrane protein (IFITM) family are ISGs that have been found to function during RSV. The absence of either IFITM1 or IFITM3 in vivo increased the pulmonary RSV viral load and enhanced weight loss, suggesting an inability to control the virus compared to WT mice [49,50]. In contrast, the overexpression of IFITM1 in either Vero or A549 cells reduced RSV infectivity by nearly 75% compared to untransduced cells [50]. The antiviral effects of IFITM1 are dependent on type I IFN, as siRNA knockdown of IFN-α rescued RSV infectivity. Additionally, in vitro administration of IFN-α/β enhanced IFITM1, IFITM2, and IFITM3 expression in RSV-infected HeLa and HEp-2 cells [51]. Finally, ISGs drive a positive feedback loop of type I IFN production, enhancing the expression of PRR signaling components including RIG-I-like receptors [52]. Type I IFN induction of ISGs such as the IFITM family are important for mediating early control of RSV. Overall, TLR and RIG-I-like receptor recognition of RSV in many innate cell populations drives early type I IFN production, promoting viral clearance and the early production of proinflammatory cytokines. 

### 2.2. Impairments in Neonatal Type I IFN Responses

Nearly all children are infected with RSV by the age of two, and age correlates with increased disease severity following natural infection [53]. Children either <6 months of age or those born prematurely are more likely to exhibit severe disease and mortality following RSV infection [54]. Additionally, premature infants (<32 weeks) have an 8.1%–13.9% higher RSV rehospitalization rate compared to full term infants [55,56]. While the reasons for enhanced RSV pathogenesis in infants are not completely clear, understanding the shortcomings of the neonatal immune response to RSV is a useful step towards developing more successful treatments and vaccines. 

Many DC subsets increase in numbers in both murine and human RSV infections [57]. Myeloid DCs and pDCs are found in high numbers in the nasal mucosa of RSV-infected individuals, potentially due to recruitment from the blood, as DC numbers in the blood subsequently decrease [58]. pDC numbers also increase substantially in mouse models of RSV infection, with recruitment detected as early as 24 hours and peaking around day 6 in the lungs [46,59]. Neonates are impaired in their initial recruitment of pDCs in response to RSV. Both the frequency and number of pDCs are reduced in neonatal mice compared to adult mice infected with RSV [59,60]. One group reported observing a nearly 15-fold difference in pDC numbers between neonates and adult mice that received RSV. Strikingly, the percentage and number of pDCs in RSV-infected neonates barely increased above that of a naïve neonatal mouse (0.26% versus 0.38%) [59]. Thus, the diminished influx of total DCs into the neonatal lung may help explain the enhanced susceptibility to RSV infection. 

Due to the antiviral role of type I IFN production by pDCs, it has been widely studied as another potential limitation in the neonatal immune response to RSV. pDCs are functionally impaired in their type I IFN production in human infants and neonatal mice. RSV-induced production of IFN-α and IFN-β in the lungs was significantly diminished in five-day-old neonatal mice compared to adults [59,61]. This impairment was observed as early as 12 hours post infection [60]. The in vivo murine data corroborates similar observations in humans. Ex vivo infection of infant cord blood cells with either RSV-A2 or RSV-Long resulted in limited IFN-α production compared to PBMCs obtained from healthy adults [62]. However, in both groups nearly all production came from pDCs. Lower levels of type 1 IFN are also observed in nasal washes of children with detectable RSV compared to either children infected with influenza virus or healthy controls [63,64,65]. Interestingly, infants produce the least amount of IFN-α, while children between one and five years old produce moderate levels of IFN-α and adults produce the highest quantity [62]. This confirms that the ability to produce type I IFNs is a function of age and suggests that inadequate type I IFN production by DCs may be a critical factor in facilitating the increased RSV disease severity observed in infants.

Many studies have also looked at the activation status and signaling capabilities of neonatal versus adult DCs to determine the mechanism driving their dysfunction. While the relative expression of RIG-I and MDA5 were normal within infant cells, IFN-α production in response to the RIG-I agonist 5’PPP-dsRNA was significantly diminished compared to adult PBMCs [62]. This impairment in RIG-I-mediated responses was even more pronounced in preterm infants, and remained through childhood, as measured in children between one and five years old. Downstream signaling components of the RIG-I- like receptor pathway, including IRF7 and IRF9, were also reduced in neonates following RSV infection [61]. These defects in early innate sensing pathways likely contribute to the increased susceptibility and pathogenesis following RSV infection in infants. Additionally, studies have found that murine neonatal DCs express lower levels of costimulation markers CD80 and CD86 in vivo early after RSV infection, resulting in impaired priming of antigen-specific CD8 T cells [60,66]. Treatment with exogenous IFN-α or IFN-β were unable to rescue DC activation or the subsequent CD8 T cell response, suggesting a functional inability to respond to type I IFN. These impairments are thought to be intrinsic to neonatal cells, as IFN-α treatment of naïve neonatal DCs was unable to upregulate the expression of either CD80 or CD86 [60]. Together, these studies suggest that infant DCs have a cell-intrinsic impairment in their type I IFN response, resulting in enhanced susceptibility to RSV.

### 2.3. Modulation of Type 1 IFN by RSV

While type I IFNs are very effective in their antiviral functions, RSV has evolved strategies to modulate and suppress the type I IFN response in infected cells. A549, 293T, and HEp-2 human epithelial cells infected with either RSV-A2 or various clinical isolates in vitro all failed to produce IFN-α above the levels induced following mock infection [67]. Similar results were observed when pDCs isolated from human PBMCs were used. RSV was also able to inhibit IFN-α production when cells were first stimulated with a TLR9 agonist, suggesting the ability of the virus to shut off ongoing type I IFN signaling. Thus, RSV effectively suppresses the hosts type I IFN response following initial infection.

The main modulators of the type I IFN response have been shown by numerous groups to be the RSV NS1 and NS2 proteins. Type I IFN modulation was first demonstrated when A549 pulmonary epithelial cells infected with a mutant virus lacking NS1 and/or NS2 (ΔNS1/ΔNS2) were found to produce higher levels of IFN-α and IFN-β mRNA and protein compared to WT RSV-A2 [11]. Similar results were observed in both primary human monocytes and blood-derived macrophages. The suppression of type I IFNs was validated by in vitro siRNA-mediated knockdown of NS1 in A549 epithelial cells, as well as an siRNA plasmid targeting NS1 delivered via a polymer carrier to WT mice in vivo [68]. Infection with a ΔNS1/NS2-RSV lacking both proteins produced higher IFN-α and IFN-β levels than the single deletions, demonstrating the cooperative functions of NS1 and NS2 [11]. Together these studies highlight the potent suppression of the type I IFN response by RSV proteins.

**Figure 1:**
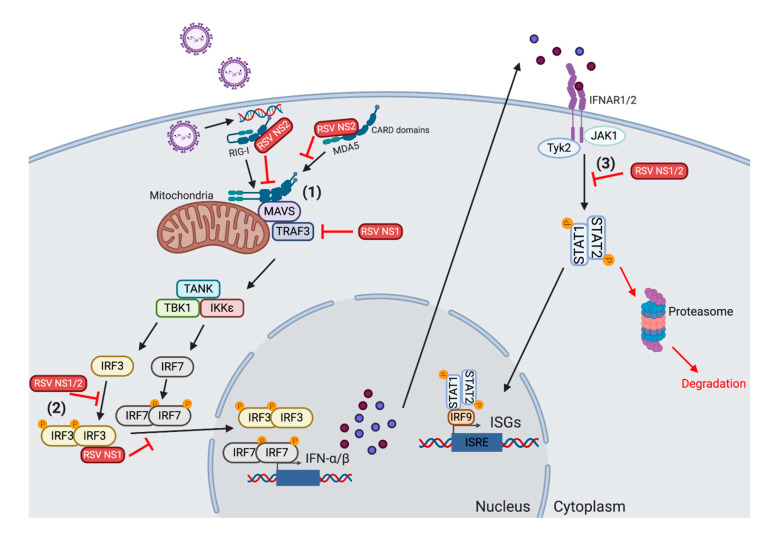
Mechanisms for respiratory syncytial virus (RSV) inhibition of type I interferon (IFN) production. (**1**) The RSV NS1 and NS2 proteins can act on many components of the type I IFN induction pathway in order to reduce the production of IFN-α/β. NS2 binds to the caspase activation and recruitment domains (CARDs) on retinoic acid-inducible gene-I-like receptor (RIG-I) and melanoma differentiation-associated protein 5 (MDA5). This blocks RIG-I from dimerizing and interacting with mitochondrial antiviral signaling (MAVS). NS1 also reduces the level of TNF receptor-associated factor (TRAF)3 inside the cell, inhibiting the subsequent activation of the downstream TANK-binding kinase 1 (TBK1)/IκB kinase (IKKε) complex. (**2**) Both NS1 and NS2 block the translocation of interferon regulatory factor (IRF)3/IRF7 into the nucleus. NS1 can bind and sequester IRF3 in the cytosol. NS1 and NS2 both inhibit the upstream phosphorylation of IRF3. (**3**) NS1 and NS2 also act on the IFN receptor (IFNAR) signaling pathway to obstruct the induction of type I IFN-mediated interferon-stimulating gene (ISG) production. NS2 reduces the levels of both total signal transducer and activator of transcription (STAT)2 and phoshorylated STAT2. The dimished activation inhibits the nuclear translocation of STAT1/STAT2, reducing the induction of ISGs. Finally, the presence of NS1 lowers the quantity of ISGs found in infected cells. Created with BioRender.com

NS1 and NS2 mediate suppression primarily by modulating the type I IFN induction pathway, as summarized in Figure 1. NS2 can interact directly with the CARD domains on RIG-I, as measured by coimmunoprecipitation experiments in 293T cells [69]. This blocks the interaction between RIG-I and MAVS, preventing the downstream induction of IFNα and IFN-β. Transfection of NS1 but not NS2 into A549 cells reduced the protein levels of TRAF3 in the cells, while infection with ΔNS1-RSV increased the levels of TRAF3 compared to WT RSV infection [70]. This suggests that NS1 can suppress type I IFNs by reducing the presence of downstream signaling targets. NS1 and NS2 can also cooperatively block translocation of IRF3 to the nucleus, as shown by the increased nuclear presence in either ΔNS1 or ΔNS2-RSV infected cells [69,71]. This occurs by preventing the upstream phosphorylation of IRF3, or binding and sequestering IRF3 to prevent downstream association [72]. The suppressive role of the RSV NS1 and NS2 proteins is specific for type I IFNs, as IL-6, IL-8, and TNF are not altered following infection with ΔNS1/2-RSV [67,73]. Thus, NS1 and NS2 act on many molecules throughout the TLR/RIG-I receptor signaling cascade, preventing infected cells from initiating early type I IFN inflammatory responses.

In addition to suppressing the induction of type I IFN, NS1 and NS2 can also alter the IFNAR signaling cascade. Transfection of epithelial cells with plasmids expressing NS2 reduced the expression of the total STAT2 and phosphorylated STAT2, while NS1 had no effect [70,74]. The effect of NS2 on STAT2 expression was recapitulated using NS2 siRNA knockdown during in vitro RSV infection [75]. The NS2-mediated reduction in STAT2 levels following RSV infection is likely due to the enhanced induction of proteasome-mediated degradation, as shown by pretreating cells with a proteasome inhibitor. [75,76]. Finally, NS1 can have a modulatory effect on the production of ISGs themselves. Oligoadenylate synthetase (OAS) levels decreased in epithelial cells modified to express NS1, NS2 or both in combination [74]. Thus, even if host cells are able to produce type I IFNs, RSV can still inhibit the activity of subsequent cytokines and ISGs by acting on downstream IFNAR signaling components.

NS1 can also modulate the activation and subsequent cytokine production by DCs. Compared to WT RSV infection, ΔNS1/2 RSV infected monocyte-derived DCs isolated from human PBMCs express lower levels of IL-6, CCL3, and TNF, as well as CD80, CD86, and CD83, markers associated with mature, activated DCs [77]. The increase in cytokine production is partially due to the known antagonism of type I IFN production in DCs by NS1 and NS2, as IFNAR2-blocking antibodies inhibit most of the cytokine upregulation. It is possible that NS1 and NS2 reduce DC activation in order to suppress the initiation of any antiviral responses following infection. Overall, the RSV NS1 and NS2 proteins obstruct both the TLR signaling pathway and subsequent IFNAR signaling to inhibit the production and function of type I IFNs.

### 2.4. Alterations in the Adaptive Immune Response by Type 1 IFNs

While the function of type I IFNs during the innate immune response to RSV is well studied, type I IFNs also play a role in shaping the subsequent adaptive response. DCs link the innate and the adaptive immune systems by priming T cells and B cells to encountered antigens through direct major histocompatibility complex (MHC)-T cell receptor (TCR)/B cell receptor (BCR) interactions, as well as secreted cytokines like type I IFNs [78]. If DCs are dysfunctional in their type I IFN production, they may prime a defective adaptive response [60,79]. In vitro studies have shown that bone marrow-derived DCs that either lack type I IFN receptor or are cultured with anti-IFNα/IFN-β antibodies fail to stimulate proliferation from CD8 and CD4 T cells, suggesting that the ability to respond and produce type I IFNs is essential for DC activation of T cells in vitro [79]. Antigen-specific T cell responses to RSV are also modulated by type I IFN production from DCs. In the absence of pDCs, a major source of type I IFN during RSV, the number of RSV M_187–195_-specific CD8 T cells and their capacity to produce IFN-γ in response to peptide stimulation are reduced compared to WT mice [45]. RSV infection can affect DC priming, as CD4 T cells exhibited reduced proliferation when cultured with RSV infected monocyte-derived DCs compared to uninfected DCs [80]. Therefore, RSV infected DCs are impaired in their ability to prime T cells, potentially due to diminished type I IFN production.

The cytokine signals produced by infected DCs can alter the polarization of T cells [78]. A Th2-biased response characterized by IL-4 producing T cells is associated with increased immunopathology during RSV infection [81,82,83]. IL-4 overexpression in vivo delayed viral clearance, while eliminating IL-4 using antibody treatment reduced weight loss compared to control mice [84,85]. In neonatal mice that normally exhibit a strong Th2-biased T cell response following secondary exposure to RSV, IFN-α treatment reduced the frequency of IL-4 producing CD4 T cells upon subsequent RSV infection [59]. The total IL-4 levels in the BAL were also reduced when mice were pretreated with IFN-α. Inhibition of a Th2 T cell response is mediated by pDCs, as adoptive transfer of adult pDCs that produce normal levels of type I IFNs is attenuated the pathological Th2 T cell response. Additionally, antibody depletion of pDC-enhanced IL-4 production by CD4 T cells in murine lungs in response to RSV [13]. Type I IFNs are vital for preventing a Th2-mediated response, and it is essential to understand the signals that promote this pathological immune response in infants. Thus, the role that DC-produced type I IFNs play in priming a productive T cell response in infants needs to be further investigated. 

While it has been less studied, type I IFNs have also been shown to modulate the humoral immune response to RSV. The absence of either MAVS or MyD88 results in reduced serum concentrations of total RSV-specific and RSV N-specific IgG and IgG2a antibodies compared to WT mice [17,61]. Fms-related tyrosine kinase 3 (Flt3) ligand, a growth factor that stimulates an increase in DC numbers rescued the defective type I IFN pathway observed in neonates and promoted increased whole-virus and RSV N-specific IgG2a 8 days post-RSV [61]. This suggests that early TLR and/or RIG-I-like receptor-mediated sensing of viral components alters the magnitude of the subsequent humoral response to RSV. 

It has also been demonstrated that type I IFNs have an effect on B cells in murine models of RSV infection. Exogenous IFN-α/β increased B cell numbers in the lungs and reduced apoptosis in cultured mature B cells as measured by terminal deoxynucleotidyl transferase dUTP nick end labeling (TUNEL) staining [86,87]. B cells exposed to IFN-α/β exhibited dose-dependent increases in CD69 and CD25 expression and increased IgM internalization, indicative of early B cell activation. Type I IFNs have also been implicated in driving IgA production in response to RSV infection [87]. Neonatal mice with low production of IFN-α and IFN-β exhibit impaired IgA production in the nasal-associated lymphoid tissue compared to age-matched controls. Furthermore, the number of B-cell activating factor (BAFF)^-^expressing B cells as well as IgA production by B cells in the nasal wash and lungs were enhanced to levels comparable with adults following pretreatment with IFN-α. This suggests that the pro-survival and activating effect of type I IFN on B cells during RSV could be driven through BAFF/a proliferation-inducing ligand (APRIL) signaling and are important for mediating protective mucosal antibody responses. Overall, RSV infection impairs the ability of DCs to prime an effective adaptive response, and this impairment is intrinsic to the neonatal immune system. Additionally, type I IFNs are fundamental for the development of a productive Th1 T cell response and a protective isotype-switched antibody response. 

## 3. Use of Type I IFNs in Therapeutic and Preventative Strategies for RSV

### 3.1. Type 1 IFN as a Therapeutic for RSV 

Approved treatment methods for RSV are limited to passive immunization with a monoclonal antibody. Palivizumab, a humanized antibody against the RSV F protein, is only administered prophylactically to high-risk infants [88]. It is expensive to administer and has a low efficacy, only preventing the rate of hospitalization due to severe RSV disease by approximately 50% [88,89]. Additionally, palivizumab is ineffective if it is administered after RSV infection has occurred, therefore therapeutics are needed to treat ongoing RSV infections [90]. Due to their strong antiviral functions both in vitro and in in vivo animal models, type I IFNs have been evaluated as a potential treatment for RSV. In mice, recombinant IFN-α treatment prior to RSV infection protected against weight loss and reduced pathology scores [91]. To test the practical application of using IFN-α as a therapeutic, IFN-α was administered three days post RSV infection. Mice that received IFN-α still exhibited reductions in viral titers, as well as significantly reduced pathology scores, as determined by histology, indicating that therapeutic IFN-α may be an efficacious treatment for RSV. 

When IFN-α treatment was tested in human clinical trials, minimal evidence was found for the effective use of type I IFNs to treat RSV. Sung et al. gave intramuscular injections of IFNα2a to RSV^+^ adults for three days and observed no difference in either viral clearance or clinical severity compared to placebo-treated individuals [92]. A similar study performed in RSV-positive infants observed no difference in either clinical signs or viral shedding between the IFNα2a treatment group and placebo controls [93]. In contrast, a recent study observed that nebulized IFN-α1b administered to children immediately following the onset of bronchiolitis symptoms reduced wheezing and coughing compared to controls receiving only minimal analgesics and antipyretics [94]. This suggests that the administration route and the class of type I IFN used may differentially affect RSV disease in humans. However, the specific viral infection causing bronchiolitis was not assessed; therefore, the effect of nebulized IFN-α1b on RSV viral clearance could not be determined. Another study administered a nasal spray of IFNα2a three days before and after experimental challenge with RSV [95]. Compared to the placebo, prophylactic administration of IFNα2a reduced both clinical scores and viral secretion in nasal washes following RSV challenge. Thus, while therapeutic administration of IFN-α may not be effective against RSV, the induction of type I IFNs may be a critical component in a successful RSV vaccine.

### 3.2. Implications for Vaccine Design

RSV has remained elusive to vaccine development efforts despite widespread research in the field. One challenge is developing a successful vaccine formulation that provides long-lasting protection without inducing immunopathology. As evidenced in this review, it will be essential to consider age-dependent differences in both the innate and adaptive immune responses to RSV. Given their role in both antiviral and proinflammatory functions, type I IFNs in particular will be critical to consider when evaluating vaccine responses in infants. If type I IFN levels can be enhanced through the use of a live-attenuated vaccine, it may prevent the increased disease severity seen in infants and young children. 

Engineered RSV viruses that have been modified to remove the NS1, NS2 or M2-2 proteins have demonstrated promising results as a vaccine in animal models of RSV. They are attenuated in vitro and in vivo, allowing them to activate RSV-specific responses without inducing WT levels of RSV replication and pathology [96,97,98]. Additionally, removal of the NS1 and NS2 proteins enhances activation of both innate and adaptive cells responding to RSV [11,77,99]. Mutant viruses lacking NS1 and/or NS2 (ΔNS1/ΔNS2) produce higher levels of IFN-α and IFN-β mRNA and protein compared to WT RSV-A2 [11]. Initial studies in seronegative chimpanzees showed reduced viral replication in the nasal passages and airways, but a similar induction of RSV-specific serum neutralizing antibodies [97]. This vaccine candidate, LID/ΔM2-2/1030s, recently underwent Phase I trial testing in seronegative children and induced increased serum neutralizing antibody titers [98,100]. However, vaccinated children also exhibited an increased rate of respiratory illness compared to placebo recipients. A similar vaccine candidate was recently tested in RSV seronegative infants in the hopes of reducing the pathogenic side effects [101]. However, while there was a greater than four-fold enhancement in neutralizing antibody titers, fever and respiratory illness still occurred in nearly 75% of subjects. Therefore, while the ΔNS1 and ΔM2-2 viruses induce strong RSV-specific immune responses, additional studies will be necessary to evaluate their safety, as well as their efficacy in a previously infected population. 

The RSV G protein has also been identified to play a role in attenuating the type I IFN response. Murine lung epithelial cells infected in vitro with a mutant RSV virus lacking the G protein (ΔG) exhibit increased levels of secreted IFN-β compared to WT RSV infection [102]. This suppression was linked to the induction of suppressor of cytokine signaling (SOCS) proteins, SOCS1 and SOCS3. SOCS1 can interact directly with JAK molecules, inhibiting their catalytic activity and subsequent type I IFN production [103,104]. SOCS1 and SOCS3 also bind to IRF7, inhibiting its phosphorylation and downstream translocation to the nucleus [105]. Normal human bronchial epithelial (NHBE) cells infected with RSV, lacking the G protein-induced lower levels of SOCS1 and SOCS3 mRNA compared to WT RSV infection [106]. This effect was mirrored by an increase in IFN-α/β production early after infection, suggesting that RSV G suppresses the type I IFN response by inducing expression of SOCS proteins. In mice, immunization with G gene-modified (Gmem) RSV, lacking expression of secreted G, protected mice from viral replication upon subsequent WT RSV challenge [107]. Thus, vaccination with ΔG RSV may enhance the antiviral type I IFN production, providing protection from RSV infection. This provides a novel target for vaccine development; however, a live-attenuated RSV vaccine lacking G has not been tested in humans.

Given the role of both NS1/2 and the G protein in the suppression of type I IFNs, modified viruses lacking these proteins may induce high levels of IFN-α and IFN-β when administered as a vaccine. This would stimulate potent antiviral mechanisms; however, this needs to be further explored in humans. While administration of recombinant IFN-α alone may not be the best approach, the generation of a vaccine that can drive the downstream production of type I IFNs is a viable option.

## 4. Conclusions

RSV continues to be the major cause of lower respiratory tract infections in children and young infants worldwide. Healthy adults exhibit only minor cold-like symptoms following infection; however, high-risk populations including infants, immunocompromised patients, and elderly individuals are at risk for severe bronchiolitis. Despite the major impact of the virus and the ongoing research worldwide, there remains only one licensed prophylactic option to combat the virus. While Palivizumab works to reduce hospitalizations in high-risk populations, the low efficacy and high cost emphasize the critical need for more options.

Type I IFNs are produced following TLR and RIG-I-like receptor recognition of RSV. They are critical mediators of viral control and the early induction of proinflammatory cytokines. Development of a productive Th1 T cell response and class-switched mucosal antibody production are both dependent on type I IFNs. This is likely driven by effective DC priming. Numerous studies support that the level of type I IFNs in nasal washes and PBMCs inversely correlate with disease severity in both adults and children. Polymorphisms in the type I IFN pathway correlate with enhanced RSV severity and longer duration of hospital stay in the intensive care unit. Additionally, infant DCs are intrinsically impaired in their production of type I IFNs, likely contributing to their association with increased RSV disease severity. Further studies into the role of IFNs may provide novel insights into the defects in the infant immune response that drive enhanced disease. A better understanding of these mechanisms will aid in the development of successful therapeutics and vaccines for adults and infants. While RSV has effective countermeasures for inhibiting the immune response, vaccines that establish a durable immune response may be able to overcome these evasion mechanisms. Thus, while therapeutic IFN-α administration may not be effective, generating a vaccine that can establish enhanced type I IFN production in a healthy individual would likely be an effective mechanism for preventing RSV infection.

## References

[1] Scheltema N.M., Gentile A., Lucion F., Nokes D.J., Munywoki P.K., Madhi S.A., Groome M.J., Cohen C., Moyes J., Thorburn K. (2017). Global respiratory syncytial virus-associated mortality in young children (RSV GOLD): A retrospective case series. Lancet Glob. Health.

[2] Nair H., Nokes D.J., Gessner B.D., Dherani M., Madhi S.A., Singleton R.J., O’Brien K.L., Roca A., Wright P.F., Bruce N. (2010). Global burden of acute lower respiratory infections due to respiratory syncytial virus in young children: A systematic review and meta-analysis. Lancet.

[3] Paramore L.C., Ciuryla V., Ciesla G., Liu L. (2004). Economic impact of respiratory syncytial virus-related illness in the US: An analysis of national databases. Pharmacoeconomics.

[4] Hall C.B., Walsh E.E., Long C.E., Schnabel K.C. (1991). Immunity to and frequency of reinfection with respiratory syncytial virus. J. Infect. Dis..

[5] Henderson F.W., Collier A.M., Clyde W.A., Denny F.W. (1979). Respiratory-syncytial-virus infections, reinfections and immunity. A prospective, longitudinal study in young children. N. Engl. J. Med..

[6] Tan L., Coenjaerts F.E.J., Houspie L., Viveen M.C., van Bleek G.M., Wiertz E.J.H.J., Martin D.P., Lemey P. (2013). The comparative genomics of human respiratory syncytial virus subgroups A and B: Genetic variability and molecular evolutionary dynamics. J. Virol..

[7] Mitra R., Baviskar P., Duncan-Decocq R.R., Patel D., Oomens A.G. (2012). The human respiratory syncytial virus matrix protein is required for maturation of viral filaments. J. Virol..

[8] Cowton V.M., McGivern D.R., Fearns R. (2006). Unravelling the complexities of respiratory syncytial virus RNA synthesis. J. Gen. Virol..

[9] Batonick M., Wertz G.W. (2011). Requirements for Human Respiratory Syncytial Virus Glycoproteins in Assembly and Egress from Infected Cells. Adv. Virol..

[10] Gan S.W., Tan E., Lin X., Yu D., Wang J., Tan G.M.Y., Vararattanavech A., Yeo C.Y., Soon C.H., Soong T.W. (2012). The small hydrophobic protein of the human respiratory syncytial virus forms pentameric ion channels. J. Biol. Chem..

[11] Spann K.M., Tran K.C., Chi B., Rabin R.L., Collins P.L. (2004). Suppression of the induction of alpha, beta, and lambda interferons by the NS1 and NS2 proteins of human respiratory syncytial virus in human epithelial cells and macrophages [corrected]. J. Virol..

[12] Goritzka M., Makris S., Kausar F., Durant L.R., Pereira C., Kumagai Y., Culley F.J., Mack M., Akira S., Johansson C. (2015). Alveolar macrophage-derived type I interferons orchestrate innate immunity to RSV through recruitment of antiviral monocytes. J. Exp. Med..

[13] Smit J.J., Rudd B.D., Lukacs N.W. (2006). Plasmacytoid dendritic cells inhibit pulmonary immunopathology and promote clearance of respiratory syncytial virus. J. Exp. Med..

[14] Hijano D.R., Vu L.D., Kauvar L.M., Tripp R.A., Polack F.P., Cormier S.A. (2019). Role of Type I Interferon (IFN) in the Respiratory Syncytial Virus (RSV) Immune Response and Disease Severity. Front. Immunol..

[15] Liu P., Jamaluddin M., Li K., Garofalo R.P., Casola A., Brasier A.R. (2007). Retinoic acid-inducible gene I mediates early antiviral response and Toll-like receptor 3 expression in respiratory syncytial virus-infected airway epithelial cells. J. Virol..

[16] Demoor T., Petersen B.C., Morris S., Mukherjee S., Ptaschinski C., De Almeida Nagata D.E., Kawai T., Ito T., Akira S., Kunkel S.L. (2012). IPS-1 signaling has a nonredundant role in mediating antiviral responses and the clearance of respiratory syncytial virus. J. Immunol..

[17] Bhoj V.G., Sun Q., Bhoj E.J., Somers C., Chen X., Torres J.P., Mejias A., Gomez A.M., Jafri H., Ramilo O. (2008). MAVS and MyD88 are essential for innate immunity but not cytotoxic T lymphocyte response against respiratory syncytial virus. Proc. Natl. Acad. Sci. USA.

[18] Goritzka M., Durant L.R., Pereira C., Salek-Ardakani S., Openshaw P.J.M., Johansson C. (2014). Alpha/beta interferon receptor signaling amplifies early proinflammatory cytokine production in the lung during respiratory syncytial virus infection. J. Virol..

[19] Foster G.R. (1997). Interferons in host defense. Semin. Liver Dis..

[20] Kumar H., Kawai T., Akira S. (2011). Pathogen recognition by the innate immune system. Int. Rev. Immunol..

[21] Kawasaki T., Kawai T. (2014). Toll-like receptor signaling pathways. Front. Immunol..

[22] Loo Y.M., Gale M. (2011). Immune signaling by RIG-I-like receptors. Immunity.

[23] Kim T.H., Lee H.K. (2014). Innate immune recognition of respiratory syncytial virus infection. BMB Rep..

[24] Murawski M.R., Bowen G.N., Cerny A.M., Anderson L.J., Haynes L.M., Tripp R.A., Kurt-Jones E.A., Finberg R.W. (2009). Respiratory syncytial virus activates innate immunity through Toll-like receptor 2. J. Virol..

[25] Kurt-Jones E.A., Popova L., Kwinn L., Haynes L.M., Jones L.P., Tripp R.A., Walsh E.E., Freeman M.W., Golenbock D.T., Anderson L.J. (2000). Pattern recognition receptors TLR4 and CD14 mediate response to respiratory syncytial virus. Nat. Immunol..

[26] Rallabhandi P., Phillips R.L., Boukhvalova M.S., Pletneva L.M., Shirey K.A., Gioannini T.L., Weiss J.P., Chow J.C., Hawkins L.D., Vogel S.N. (2012). Respiratory syncytial virus fusion protein-induced toll-like receptor 4 (TLR4) signaling is inhibited by the TLR4 antagonists Rhodobacter sphaeroides lipopolysaccharide and eritoran (E5564) and requires direct interaction with MD-2. mBio.

[27] Marr N., Turvey S.E. (2012). Role of human TLR4 in respiratory syncytial virus-induced NF-kappaB activation, viral entry and replication. Innate Immun..

[28] Awomoyi A.A., Rallabhandi P., Pollin T.I., Lorenz E., Sztein M.B., Boukhvalova M.S., Hemming V.G., Blanco J.C.G., Vogel S.N. (2007). Association of TLR4 polymorphisms with symptomatic respiratory syncytial virus infection in high-risk infants and young children. J. Immunol..

[29] Caballero M.T., Serra M.E., Acosta P.L., Marzec J., Gibbons L., Salim M., Rodriguez A., Reynaldi A., Garcia A., Bado D. (2015). TLR4 genotype and environmental LPS mediate RSV bronchiolitis through Th2 polarization. J. Clin. Invest..

[30] Tulic M.K., Hurrelbrink R.J., Prele C.M., Laing I.A., Upham J.W., Le Souef P., Sly P.D., Holt P.G. (2007). TLR4 polymorphisms mediate impaired responses to respiratory syncytial virus and lipopolysaccharide. J. Immunol..

[31] Haynes L.M., Moore D.D., Kurt-Jones E.A., Finberg R.W., Anderson L.J., Tripp R.A. (2001). Involvement of toll-like receptor 4 in innate immunity to respiratory syncytial virus. J. Virol..

[32] Loo Y.M., Fornek J., Crochet N., Bajwa G., Perwitasari O., Martinez-Sobrido L., Akira S., Gill M.A., Garcia-Sastre A., Katze M.G. (2008). Distinct RIG-I and MDA5 signaling by RNA viruses in innate immunity. J. Virol..

[33] Bitko V., Musiyenko A., Bayfield M.A., Maraia R.J., Barik S. (2008). Cellular La protein shields nonsegmented negative-strand RNA viral leader RNA from RIG-I and enhances virus growth by diverse mechanisms. J. Virol..

[34] Asgari S., Schlapbach L.J., Anchisi S., Hammer C., Bartha I., Junier T., Mottet-Osman G., Posfay-Barbe K.M., Longchamp D., Stocker M. (2017). Severe viral respiratory infections in children with IFIH1 loss-of-function mutations. Proc. Natl. Acad. Sci. USA.

[35] Belgnaoui S.M., Paz S., Hiscott J. (2011). Orchestrating the interferon antiviral response through the mitochondrial antiviral signaling (MAVS) adapter. Curr. Opin. Immunol..

[36] Seth R.B., Sun L., Ea C.K., Chen Z.J. (2005). Identification and characterization of MAVS, a mitochondrial antiviral signaling protein that activates NF-kappaB and IRF 3. Cell.

[37] Barik S. (2013). Respiratory syncytial virus mechanisms to interfere with type 1 interferons. Curr. Top. Microbiol. Immunol..

[38] Makris S., Bajorek M., Culley F.J., Goritzka M., Johansson C. (2016). Alveolar Macrophages Can Control Respiratory Syncytial Virus Infection in the Absence of Type I Interferons. J. Innate Immun..

[39] Jewell N.A., Vaghefi N., Mertz S.E., Akter P., Peebles R.S., Bakaletz L.O., Durbin R.K., Flano E., Durbin J.E. (2007). Differential type I interferon induction by respiratory syncytial virus and influenza a virus in vivo. J. Virol..

[40] Hornung V., Schlender J., Guenthner-Biller M., Rothenfusser S., Endres S., Conzelmann K.K., Hartmann G. (2004). Replication-dependent potent IFN-alpha induction in human plasmacytoid dendritic cells by a single-stranded RNA virus. J. Immunol..

[41] Johnson T.R., Johnson C.N., Corbett K.S., Edwards G.C., Graham B.S. (2011). Primary human mDC1, mDC2, and pDC dendritic cells are differentially infected and activated by respiratory syncytial virus. PLoS ONE.

[42] Boogaard I., van Oosten M., van Rijt L.S., Muskens F., Kimman T.G., Lambrecht B.N., Buisman A.M. (2007). Respiratory syncytial virus differentially activates murine myeloid and plasmacytoid dendritic cells. Immunology.

[43] Schijf M.A., Lukens M.V., Kruijsen D., van Uden N.O.P., Garssen J., Coenjaerts F.E.J., van’t Land B., van Bleek G.M. (2013). Respiratory syncytial virus induced type I IFN production by pDC is regulated by RSV-infected airway epithelial cells, RSV-exposed monocytes and virus specific antibodies. PLoS ONE.

[44] Kumagai Y., Takeuchi O., Kato H., Kumar H., Matsui K., Morii E., Aozasa K., Kawai T., Akira S. (2007). Alveolar macrophages are the primary interferon-alpha producer in pulmonary infection with RNA viruses. Immunity.

[45] Kim T.H., Oh D.S., Jung H.E., Chang J., Lee H.K. (2019). Plasmacytoid Dendritic Cells Contribute to the Production of IFN-beta via TLR7-MyD88-Dependent Pathway and CTL Priming during Respiratory Syncytial Virus Infection. Viruses.

[46] Wang H., Peters N., Schwarze J. (2006). Plasmacytoid dendritic cells limit viral replication, pulmonary inflammation, and airway hyperresponsiveness in respiratory syncytial virus infection. J. Immunol..

[47] Crisler W.J., Lenz L.L. (2018). Crosstalk between type I and II interferons in regulation of myeloid cell responses during bacterial infection. Curr. Opin. Immunol..

[48] Schoggins J.W., MacDuff D.A., Imanaka N., Gainey M.D., Shrestha B., Eitson J.L., Mar K.B., Richardson R.B., Ratushny A.V., Litvak V. (2014). Pan-viral specificity of IFN-induced genes reveals new roles for cGAS in innate immunity. Nature.

[49] Everitt A.R., Clare S., McDonald J.U., Kane L., Harcourt K., Ahras M., Lall A., Hale C., Rodgers A., Young D.B. (2013). Defining the range of pathogens susceptible to Ifitm3 restriction using a knockout mouse model. PLoS ONE.

[50] Smith S.E., Busse D.C., Binter S., Weston S., Diaz Soria C., Laksono B.M., Clare S., Van Nieuwkoop S., Van den Hoogen B.G., Clement M. (2019). Interferon-Induced Transmembrane Protein 1 Restricts Replication of Viruses That Enter Cells via the Plasma Membrane. J. Virol..

[51] Zhang W., Zhang L., Zan Y., Du N., Yang Y., Tien P. (2015). Human respiratory syncytial virus infection is inhibited by IFN-induced transmembrane proteins. J. Gen. Virol..

[52] Kang D.C., Gopalkrishnan R.V., Wu Q., Jankowsky E., Pyle A.N., Fisher P.B. (2002). mda-5: An interferon-inducible putative RNA helicase with double-stranded RNA-dependent ATPase activity and melanoma growth-suppressive properties. Proc. Natl. Acad. Sci. USA.

[53] Sommer C., Resch B., Simoes E.A. (2011). Risk factors for severe respiratory syncytial virus lower respiratory tract infection. Open Microbiol. J..

[54] Rodriguez D.A., Rodriguez-Martinez C.E., Cardenas A.C., Quilaguy I.E., Mayorga L.Y., Falla L.M., Nino G. (2014). Predictors of severity and mortality in children hospitalized with respiratory syncytial virus infection in a tropical region. Pediatr. Pulmonol..

[55] Stevens T.P., Sinkin R.A., Hall C.B., Maniscalco W.M., McConnochie K.M. (2000). Respiratory syncytial virus and premature infants born at 32 weeks’ gestation or earlier: Hospitalization and economic implications of prophylaxis. Arch. Pediatr. Adolesc. Med..

[56] (1998). Prevention of respiratory syncytial virus infections: Indications for the use of palivizumab and update on the use of RSV-IGIV. American Academy of Pediatrics Committee on Infectious Diseases and Committee of Fetus and Newborn. Pediatrics.

[57] Beyer M., Bartz H., Horner K., Doths S., Koerner-Rettberg C., Schwarze J. (2004). Sustained increases in numbers of pulmonary dendritic cells after respiratory syncytial virus infection. J. Allergy Clin. Immunol..

[58] Gill M.A., Palucka A.K., Barton T., Ghaffar F., Jafri H., Banchereau J., Ramilo O. (2005). Mobilization of plasmacytoid and myeloid dendritic cells to mucosal sites in children with respiratory syncytial virus and other viral respiratory infections. J. Infect. Dis..

[59] Cormier S.A., Shrestha B., Saravia J., Lee G.I., Shen L., DeVincenzo J.P., Kim Y., You D. (2014). Limited type I interferons and plasmacytoid dendritic cells during neonatal respiratory syncytial virus infection permit immunopathogenesis upon reinfection. J. Virol..

[60] Lau-Kilby A.W., Turfkruyer M., Kehl M., Yang L., Buchholz U.J., Hickey K., Malloy A.M.W. (2019). Type I IFN ineffectively activates neonatal dendritic cells limiting respiratory antiviral T-cell responses. Mucosal Immunol..

[61] Remot A., Descamps D., Jouneau L., Laubreton D., Dubuquoy C., Bouet S., Lecardonnel J., Rebours E., Petit-Camurdan A., Riffault S. (2016). Flt3 ligand improves the innate response to respiratory syncytial virus and limits lung disease upon RSV reexposure in neonate mice. Eur. J. Immunol..

[62] Marr N., Wang T.I., Kam S.H.Y., Hu Y.S., Sharma A.A., Lam A., Markowski J., Solimano S., Lavoie P.M., Turvey S.E. (2014). Attenuation of respiratory syncytial virus-induced and RIG-I-dependent type I IFN responses in human neonates and very young children. J. Immunol..

[63] McIntosh K. (1978). Interferon in nasal secretions from infants with viral respiratory tract infections. J. Pediatr..

[64] Hall C.B., Douglas R.G., Simons R.L., Geiman J.M. (1978). Interferon production in children with respiratory syncytial, influenza, and parainfluenza virus infections. J. Pediatr..

[65] Isaacs D. (1989). Production of interferon in respiratory syncytial virus bronchiolitis. Arch. Dis. Child..

[66] Ruckwardt T.J., Malloy A.M., Morabito K.M., Graham B.S. (2014). Quantitative and qualitative deficits in neonatal lung-migratory dendritic cells impact the generation of the CD8+ T cell response. PLoS Pathog..

[67] Schlender J., Hornung V., Finke S., Gunthner-Biller M., Marozin S., Brzozka K., Moghim S., Endres S., Hartmann G., Conzelmann K.K. (2005). Inhibition of toll-like receptor 7- and 9-mediated alpha/beta interferon production in human plasmacytoid dendritic cells by respiratory syncytial virus and measles virus. J. Virol..

[68] Zhang W., Yang H., Kong X., Mohapatra S., San Juan-Vergara H., Hellermann G., Behera S., Singam R., Lockey R.F., Mohapatra S.S. (2005). Inhibition of respiratory syncytial virus infection with intranasal siRNA nanoparticles targeting the viral NS1 gene. Nat. Med..

[69] Ling Z., Tran K.C., Teng M.N. (2009). Human respiratory syncytial virus nonstructural protein NS2 antagonizes the activation of beta interferon transcription by interacting with RIG-I. J. Virol..

[70] Swedan S., Musiyenko A., Barik S. (2009). Respiratory syncytial virus nonstructural proteins decrease levels of multiple members of the cellular interferon pathways. J. Virol..

[71] Spann K.M., Tran K.C., Collins P.L. (2005). Effects of nonstructural proteins NS1 and NS2 of human respiratory syncytial virus on interferon regulatory factor 3, NF-kappaB, and proinflammatory cytokines. J. Virol..

[72] Ren J., Liu T., Pang L., Li K., Garofalo R.P., Casola A., Bao X. (2011). A novel mechanism for the inhibition of interferon regulatory factor-3-dependent gene expression by human respiratory syncytial virus NS1 protein. J. Gen. Virol..

[73] Hastie M.L., Headlam M.J., Patel N.B., Bukreyev A.B., Buchholz U.J., Dave K.A., Norris E.L., Wright C.L., Spann K.M., Collins P.L. (2012). The human respiratory syncytial virus nonstructural protein 1 regulates type I and type II interferon pathways. Mol. Cell. Proteom..

[74] Lo M.S., Brazas R.M., Holtzman M.J. (2005). Respiratory syncytial virus nonstructural proteins NS1 and NS2 mediate inhibition of Stat2 expression and alpha/beta interferon responsiveness. J. Virol..

[75] Ramaswamy M., Shi L., Varga S.M., Barik S., Behlke M.A., Look D.C. (2006). Respiratory syncytial virus nonstructural protein 2 specifically inhibits type I interferon signal transduction. Virology.

[76] Ramaswamy M., Shi L., Monick M.M., Hunninghake G.W., Look D.C. (2004). Specific inhibition of type I interferon signal transduction by respiratory syncytial virus. Am. J. Respir. Cell Mol. Biol..

[77] Munir S., Nouen C.L., Luongo C., Buchholz U.J., Collins P.L., Bukreyev A. (2008). Nonstructural proteins 1 and 2 of respiratory syncytial virus suppress maturation of human dendritic cells. J. Virol..

[78] Reis e Sousa C. (2006). Dendritic cells in a mature age. Nat. Rev. Immunol..

[79] Montoya M., Schiavoni G., Mattei F., Gresser I., Belardelli F., Borrow P., Tough D.F. (2002). Type I interferons produced by dendritic cells promote their phenotypic and functional activation. Blood.

[80] Guerrero-Plata A., Casola A., Suarez G., Yu X., Spetch L., Peeples M.E., Garofalo R.P. (2006). Differential response of dendritic cells to human metapneumovirus and respiratory syncytial virus. Am. J. Respir. Cell Mol. Biol..

[81] Roman M., Calhoun W.J., Hinton K.L., Avendano L.F., Simon V., Escobar A.M., Gaggero A., Diaz P.V. (1997). Respiratory syncytial virus infection in infants is associated with predominant Th-2-like response. Am. J. Respir. Crit. Care Med..

[82] Bendelja K., Gagro A., Bace A., Lokar-Kolbas R., Krsulovic-Hresic V., Drazenovic V., Mlinaric-Galinovic G., Rabatic S. (2000). Predominant type-2 response in infants with respiratory syncytial virus (RSV) infection demonstrated by cytokine flow cytometry. Clin. Exp. Immunol..

[83] Nenna R., Fedele G., Frassanito A., Petrarca L., Di Mattia G., Pierangeli A., Scagnolari C., Papoff P., Schiavoni I., Leone P. (2020). Increased T-helper Cell 2 Response in Infants With Respiratory Syncytial Virus Bronchiolitis Hospitalized Outside Epidemic Peak. Pediatr. Infect. Dis. J..

[84] Fischer J.E., Johnson J.E., Kuli-Zade R.K., Johnson T.R., Aung S., Parker R.A., Graham B.S. (1997). Overexpression of interleukin-4 delays virus clearance in mice infected with respiratory syncytial virus. J. Virol..

[85] Tang Y.W., Graham B.S. (1994). Anti-IL-4 treatment at immunization modulates cytokine expression, reduces illness, and increases cytotoxic T lymphocyte activity in mice challenged with respiratory syncytial virus. J. Clin. Invest..

[86] Braun D., Caramalho I., Demengeot J. (2002). IFN-alpha/beta enhances BCR-dependent B cell responses. Int. Immunol..

[87] Hijano D.R., Siefker D.T., Shrestha B., Jaligama S., Vu L.D., Tillman H., Finkelstein D., Saravia J., You D., Cormier S.A. (2018). Type I Interferon Potentiates IgA Immunity to Respiratory Syncytial Virus Infection During Infancy. Sci. Rep..

[88] Anderson E.J., Carosone-Link P., Yogev R., Yi J., Simoes E.A.F. (2017). Effectiveness of Palivizumab in High-risk Infants and Children: A Propensity Score Weighted Regression Analysis. Pediatr. Infect. Dis. J..

[89] Narbona-Lopez E., Uberos J., Checa-Ros A., Rodriguez-Belmonte R., Munoz-Hoyos A. (2018). Prevention of syncytial respiratory virus infection with palivizumab: Descriptive and comparative analysis after 12 years of use. Minerva Pediatr..

[90] Alansari K., Toaimah F.H., Almatar D.H., El Tatawy L.A., Davidson B.L., Qusad M.I.M. (2019). Monoclonal Antibody Treatment of RSV Bronchiolitis in Young Infants: A Randomized Trial. Pediatrics.

[91] Guerrero-Plata A., Baron S., Poast J.S., Adegboyega P.A., Casola A., Garofalo R.P. (2005). Activity and regulation of alpha interferon in respiratory syncytial virus and human metapneumovirus experimental infections. J. Virol..

[92] Sung R.Y., Yin J., Oppenheimer S.J., Tam J.S., Lau J. (1993). Treatment of respiratory syncytial virus infection with recombinant interferon alfa-2a. Arch. Dis Child..

[93] Chipps B.E., Sullivan W.F., Portnoy J.M. (1993). Alpha-2A-interferon for treatment of bronchiolitis caused by respiratory syncytial virus. Pediatr. Infect. Dis. J..

[94] Chen L., Shi M., Deng Q., Liu W., Li Q., Ye P., Yu X., Zhang B., Xu Y., Li X. (2020). A multi-center randomized prospective study on the treatment of infant bronchiolitis with interferon alpha1b nebulization. PLoS ONE.

[95] Higgins P.G., Barrow G.I., Tyrrell D.A., Isaacs D., Gauci C.L. (1990). The efficacy of intranasal interferon alpha-2a in respiratory syncytial virus infection in volunteers. Antivir. Res..

[96] Jin H., Zhou H., Cheng X., Tang R., Munoz M., Nguyen N. (2000). Recombinant respiratory syncytial viruses with deletions in the NS1, NS2, SH, and M2-2 genes are attenuated in vitro and in vivo. Virology.

[97] Teng M.N., Whitehead S.S., Bermingham A., St. Claire M., Elkins W.R., Murphy B.R., Collins P.L. (2000). Recombinant respiratory syncytial virus that does not express the NS1 or M2-2 protein is highly attenuated and immunogenic in chimpanzees. J. Virol..

[98] Teng M.N., Mejias A., Ramilo O., Peeples M.E. (2019). Live Attenuated Vaccine with a Stabilized Mutation and Gene Deletion for Prevention of Respiratory Syncytial Virus Disease in Young Children. J. Infect. Dis..

[99] Munir S., Hillyer P., Le Nouen C., Buchholz U.J., Rabin R.L., Collins P.L., Bukreyev A. (2011). Respiratory syncytial virus interferon antagonist NS1 protein suppresses and skews the human T lymphocyte response. PLoS Pathog..

[100] McFarland E.J., Karron R.A., Muresan P., Cunningham C.K., Valentine M.E., Perlowski C., Thumar B., Gnanashanmugam D., Siberry G.K., Schappell E. (2018). Live-Attenuated Respiratory Syncytial Virus Vaccine Candidate With Deletion of RNA Synthesis Regulatory Protein M2-2 is Highly Immunogenic in Children. J. Infect. Dis..

[101] McFarland E.J., Karron R.A., Muresan P., Cunningham C.K., Perlowski C., Libous J., Oliva J., Jean-Philippe P., Moye J., Schappell E. (2020). Live-attenuated respiratory syncytial virus vaccine with M2-2 deletion and with SH non-coding region is highly immunogenic in children. J. Infect. Dis..

[102] Moore E.C., Barber J., Tripp R.A. (2008). Respiratory syncytial virus (RSV) attachment and nonstructural proteins modify the type I interferon response associated with suppressor of cytokine signaling (SOCS) proteins and IFN-stimulated gene-15 (ISG15). Virol. J..

[103] Starr R., Willson T.A., Viney E.M., Murray L.J.L., Rayner J.R., Jenkins B.J., Gonda T.J., Alexander W.S., Metcalf D., Nicola N.A. (1997). A family of cytokine-inducible inhibitors of signalling. Nature.

[104] Croker B.A., Kiu H., Nicholson S.E. (2008). SOCS regulation of the JAK/STAT signalling pathway. Semin. Cell Dev. Biol..

[105] Yu C.F., Peng W.M., Schlee M., Barchet W., Eis-Hubinger A.M., Kolanus W., Geyer M., Schmitt S., Steinhagen F., Oldenburg J. (2018). SOCS1 and SOCS3 Target IRF7 Degradation To Suppress TLR7-Mediated Type I IFN Production of Human Plasmacytoid Dendritic Cells. J. Immunol..

[106] Oshansky C.M., Krunkosky T.M., Barber J., Jones L.P., Tripp R.A. (2009). Respiratory syncytial virus proteins modulate suppressors of cytokine signaling 1 and 3 and the type I interferon response to infection by a toll-like receptor pathway. Viral Immunol..

[107] Maher C.F., Hussell T., Blair E., Ring C.J., Openshaw P.J. (2004). Recombinant respiratory syncytial virus lacking secreted glycoprotein G is attenuated, non-pathogenic but induces protective immunity. Microbes Infect..

